# Comparative study of ^18^F-DCFPyL PET/CT and ^99m^Tc-MDP SPECT/CT bone imaging for the detection of bone metastases in prostate cancer

**DOI:** 10.3389/fmed.2023.1201977

**Published:** 2023-07-31

**Authors:** Xiongjian Hu, Yiming Cao, Bin Ji, Min Zhao, Qiang Wen, Bin Chen

**Affiliations:** Department of Nuclear Medicine, China-Japan Union Hospital of Jilin University, Changchun, China

**Keywords:** ^18^F-DCFPyL, bone scan, prostate-specific membrane antigen, bone metastasis, prostate cancer

## Abstract

**Purpose:**

This study aimed to compare the diagnostic efficiency of ^18^F-DCFPyL PET/CT imaging and ^99m^Tc-MDP SPECT/CT bone imaging for the detection of bone metastases in prostate cancer.

**Methods:**

A retrospective analysis was conducted on 31 patients with confirmed prostate cancer between September 2020 and September 2022 at China-Japan Union Hospital of Jilin University. All patients underwent ^18^F-DCFPyL PET/CT and ^99m^Tc-MDP SPECT/CT bone imaging. The gold standard was the pathology or Best Valuable Comparator (BVC) result based on clinical follow-up. Diagnostic performance indicators, including sensitivity, specificity, accuracy, positive predictive value (PPV), and negative predictive value (NPV), were analyzed at both the patient and lesion levels. The paired sample chi-square test was used to compare the two imaging methods. Receiver operating characteristic (ROC) curves were plotted, and the area under the curve (AUC) was calculated for each method. The AUC values were compared using the *Z*-test, and a *p*-value < 0.05 was considered statistically significant.

**Results:**

Of the 31 prostate cancer patients, 18 were diagnosed with bone metastases, with a total of 84 bone metastatic lesions. At the patient level, ^18^F-DCFPyL PET/CT imaging showed superior diagnostic performance compared to ^99m^Tc-MDP SPECT/CT bone imaging in all indicators: sensitivity (100% vs. 77.8%, *p* < 0.01), specificity (92.3% vs. 69.2%, *p* < 0.05), accuracy (96.8% vs. 74.2%, *p* < 0.01), PPV (94.7% vs. 77.8%, *p* < 0.01), and NPV (100% vs. 69.2%, *p* < 0.01). The AUC values for ^18^F-DCFPyL PET/CT imaging and ^99m^Tc-MDP SPECT/CT bone imaging were 0.962 and 0.735 (*Z* = 2.168, *p* < 0.05). At the lesion level, ^18^F-DCFPyL PET/CT imaging showed superior diagnostic performance compared to ^99m^Tc-MDP SPECT/CT bone imaging in all indicators: sensitivity (97.6% vs. 72.6%, *p* < 0.01), specificity (95.7% vs. 73.9%, *p* < 0.01), accuracy (97.2% vs. 72.9%, *p* < 0.01), PPV (98.8% vs. 91.0%, *p* < 0.01), and NPV (91.7% vs. 42.5%, *p* < 0.01). The AUC values for ^18^F-DCFPyL PET/CT imaging and ^99m^Tc-MDP SPECT/CT bone imaging were 0.966 and 0.733 (*Z* = 3.541, *p* < 0.001).

**Conclusion:**

Compared with ^99m^Tc-MDP SPECT/CT bone imaging, ^18^F-DCFPyL PET/CT imaging demonstrated higher diagnostic efficiency for bone metastases in prostate cancer, and it can more accurately determine the presence of bone metastases. It is an important supplement to imaging examination for prostate cancer patients and has great potential and broad application prospects.

## Introduction

Prostate cancer accounts for 7.3% of new malignant tumors worldwide in 2020 and is one of the most common malignant tumors in middle-aged and elderly men, posing a serious threat to male life and health (1, 2). However, due to the incomplete popularization of tumor screening and insufficient attention to health checkups, the overall staging of prostate cancer patients in developing countries is later than that in developed countries, resulting in a poorer overall prognosis for patients. Compared with other organ tissues, prostate cancer cells have a greater affinity for bone tissue, with over 3/4 of prostate cancer patients will develop bone metastasis and about 50% of patients having bone metastasis at the initial diagnosis (3, 4). Currently, commonly used imaging methods for diagnosing prostate cancer bone metastasis include X-ray, CT, MRI, and ^99m^Tc-MDP whole-body bone imaging. Among them, ^99m^Tc-MDP whole-body bone imaging is widely used in clinical practice due to its low price and ability to image the entire body in a single examination, making it the preferred imaging method for screening prostate cancer bone metastasis (5). With the development of imaging technology and the advent of new specific imaging agents, molecular imaging techniques represented by PET/CT are playing an increasingly important role in the precise diagnosis and treatment of malignant tumors, with Prostate-specific membrane antigen (PSMA) as a targeted molecule receiving much attention in prostate cancer diagnosis and treatment (6). Compared with radiopharmaceuticals labeled with ^68^Ga, the ^18^F-labeled PSMA imaging agent that has been clinically applied in recent years has the advantages of high synthesis rate, long half-life, and high image quality. Studies have shown that it has higher sensitivity and specificity in diagnosing prostate cancer lesions and is of significant value in initial diagnosis, staging, and therapeutic evaluation of prostate cancer (6–9). ^18^F-DCFPyL is a second-generation urea-based PSMA small-molecule inhibitor. Compared with the first-generation imaging agent ^18^F-DCFBC, its tumor affinity is nearly five times higher (9). At the same time, studies have shown that ^18^F-DCFPyL PET/CT imaging has a lower false positive rate in diagnosing bone metastasis in prostate cancer, especially in the ribs, scapula, and clavicle, compared with the more widely used ^18^F-PSMA-1007 in clinical practice (10). However, there are currently no studies comparing the diagnostic efficacy of ^18^F-DCFPyL PET/CT imaging and ^99m^Tc-MDP SPECT/CT bone imaging in prostate cancer bone metastasis. Based on this, this study retrospectively collected imaging and clinical data of prostate cancer patients who underwent both ^18^F-DCFPyL PET/CT and ^99m^Tc-MDP SPECT/CT bone imaging during the same period and further compared and analyzed the diagnostic efficacy of the two imaging methods in diagnosing prostate cancer bone metastasis, in order to provide assistance for early diagnosis and personalized treatment in clinical practice.

## Patients and methods

### Patient population and selection

This study enrolled 31 patients with histologically confirmed prostate cancer at the China-Japan Union Hospital of Jilin University between September 2020 and September 2022. All patients underwent ^99m^Tc-MDP SPECT/CT bone imaging and ^18^F-DCFPyL PET/CT imaging during the same period. The inclusion criteria were: (1) clinical diagnosis of prostate cancer, (2) the time interval between the two imaging methods did not exceed 1 month, and no changes were made to the clinical treatment plan during this period, and (3) complete general clinical data of the patients were available. Exclusion criteria were: (1) combination with other primary malignant tumors, (2) lack of clinical data or loss to follow-up. The study and application of the radioactive drug ^18^F-DCFPyL had been approved by the independent ethics committee of the China-Japan Union Hospital of Jilin University. After obtaining a comprehensive understanding of the imaging principles, radiation dose, safety, and other information, all patients provided written informed consent.

### Imaging protocol

MDP was radiolabeled with 555–925 MBq ^99m^Tc and then administer edvia a single intravenous bolus injection into the arm, followed by a 10-mL saline flush. Image acquisition was performed using dual-head SPECT/CT (Precedence; Philips Healthcare) at 3 h after injection. The patients were positioned in a supine position, and a low-energy parallel-hole collimator was used during the scanning process. They remained motionless in the same position throughout the scan. Anterior and posterior whole-body planar imaging were obtained, with photopeak set at 140 keV, a symmetrical of 20% window, a collection matrix of 256 × 1,024 pixels, and a scan speed of 20 cm/min. Subsequently, CT images were acquired for suspected metastatic areas, with a slice thickness of 2.0 mm, current 100 mAs, and a voltage of 130 kV. Immediately after the CT scan, SPECT tomographic imaging is performed, with one frame per 6° and a collection matrix of 64 × 64. During image acquisition, the detectors were positioned as close to the patient’s body surface as possible. After image acquisition, SPECT/CT image reconstruction was performed using the Extended Brilliance Workspace (Astonish SPECT algorithm, Philips Healthcare).

Our institution’s ^18^F-DCFPyL PET/CT protocol complies with the OSPREY trial and the FDA-approved PYLARIFY package insert. ^18^F-DCFPyL was injected through the elbow vein at a dose of 333–481 MBq. Image acquisition was performed using PET/CT scanner (uMI 780; United Imaging Healthcare) at 1 h after injection. Patients were in the supine position with their hands raised, and the scanning range was from the top of the head to the base of the thigh, with limb scanning added if necessary. CT scanning was performed first with the following parameters: voltage 120 kV, current 100 mAs, thickness 0.5 mm, and acquisition matrix 512 × 512. PET data were acquired using a 3D acquisition mode with a rate of 120 s per frame. PET images were corrected for attenuation using CT data (CTAC) and reconstructed using ordered subset expectation maximization (OSEM). All PET/CT scan images were processed using the uWS-CT software (United Imaging Healthcare, Shanghai, China) for image fusion and analysis.

### Image analysis

Two experienced nuclear medicine physicians independently reviewed the images in a blinded manner, without providing other radiological examination results or relevant clinical data. The diagnostic results were determined based on the consistency principle, and the initial diagnosis for the two imaging methods was obtained. The specific location and number of metastatic lesions were recorded. Patients with >5 bone metastases or multiple bone metastases that were fused and difficult to distinguish, making it difficult to accurately count the number of bone metastases, were defined as having extensive metastasis.

The study made a final diagnosis of bone metastasis in prostate cancer based on pathological or Best Valuable Comparator (BVC) results (11). BVC results are obtained by continuously following up patients for 6 months and using the dynamic changes in clinical symptoms, imaging examination results, PSA levels, and the above results as the standard for diagnosing bone metastasis in prostate cancer. Patients who meet two or more of the following conditions can be diagnosed with prostate cancer bone metastasis based on BVC results: (1) ≥2 imaging examinations indicate the presence of bone metastases; (2) there are signs of bone pain in the patient’s clinical symptoms, and imaging examination results indicate that there are bone metastatic lesions in the site of bone pain, and symptoms can be relieved after clinical treatment; (3) The bone metastatic lesions indicated by imaging examinations showed a decrease in lesion volume or reduced activity after clinical treatment; (4) PSA ≥ 100 ng/mL indicates distant metastasis (12).

### Statistical analysis

Statistical analysis of the data was performed using SPSS 25.0 and MedCalc 20.0 software. Prostate cancer patients were divided into two groups, those with and without bone metastasis, based on pathological or BVC results. The number of bone metastases in prostate cancer was separately counted using ^99m^Tc-MDP SPECT/CT bone imaging and ^18^F-DCFPyL PET/CT imaging at both patient and lesion levels. For patients with extensive bone metastasis, only patient-level analysis was conducted without further analysis at the lesion level. Inter-observer agreement was evaluated at both the patient and lesion levels using Cohen’s kappa coefficients. The diagnostic performance indices, including sensitivity, specificity, accuracy, positive predictive value (PPV), and negative predictive value (NPV), were calculated for both imaging methods. The paired sample chi-square test (McNemar test) was used to compare the above indices of the two methods. Receiver operating characteristic (ROC) curves were plotted for both imaging methods, and the area under the curve (AUC) was calculated for each. The *Z*-test was used to compare the AUC of the two methods to evaluate their diagnostic performance. A difference with *p* < 0.05 was considered statistically significant.

## Results

A total of 31 patients with prostate cancer, diagnosed based on pathology or BVC results, included 18 patients with bone metastasis. The patients had an average age of 67.83 ± 6.65 years (range: 54–78 years), and the time interval between the ^18^F-DCFPyL PET/CT and ^99m^Tc-MDP SPECT/CT imaging examinations was 10.6 ± 3.8 days (range: 1–26 days). Among the 18 patients, 5 had extensive bone metastasis, while the remaining 13 patients were diagnosed with a total of 84 bone metastatic lesions. The probabilities of bone metastasis occurring in the pelvis, spine, ribs, skull, and limbs were 72.2, 44.4, 38.9, 5.6, and 16.6%, respectively, with spine metastasis most commonly occurring in the lumbar vertebrae. The patients exhibited a mean serum t-PSA level of 70.88 ± 28.6 ng/mL, ranging from 0.15 to 372.08 ng/mL. Notably, 18 patients (58.1%) displayed a t-PSA level exceeding 20 ng/mL. In Table 1, the distribution of Gleason scores among the patients was as follows: 7 patients (22.6%) had a score of ≤6, 8 patients (25.8%) had a score of 7, and 18 patients (51.6%) had a score ≥ 8. According to the D’Amico risk stratification, 26 patients (83.9%) were classified as intermediate to high risk. High inter-observer agreement was observed for both patient-level and lesion-level analyses, with kappa values of 0.89 (95% CI 0.73–0.94) and 0.86 (95% CI 0.70–0.92) respectively.

**Table 1:** General clinical data of patients (*n* = 31).

Patient characteristics	*N* (%)
**Age (years)**	
<60	4 (12.9%)
60~70	16 (51.6%)
70~80	10 (32.3%)
>80	1 (3.2%)
**Purpose of examination**	
Initial diagnosis	10 (32.3%)
Staging	8 (25.8%)
Therapeutic evaluation	13 (41.9%)
**t-PSA level (ng/ml)**	
<10	8 (25.8%)
10~20	5 (16.1%)
>20	18 (58.1%)
**Gleason score**	
≤6	7 (22.6%)
=7	8 (25.8%)
≥8	16 (51.6%)

Based on patient-level analysis, 19 patients were found to have bone metastasis based on ^18^F-DCFPyL PET/CT imaging. Of these, one patient exhibited notable ^18^F-DCFPyL uptake in the right sacroiliac joint, which was confirmed by biopsy as degenerative joint disease, while the remaining 18 patients who tested positive for ^18^F-DCFPyL PET/CT imaging were confirmed by pathology or BVC to have bone metastatic lesions. Based on lesion-level analysis, 83 lesions exhibited varying degrees of ^18^F-DCFPyL uptake on PET/CT imaging. One lesion was determined to be a false positive result (degenerative joint disease in the right sacroiliac joint) based on pathology or BVC results. Additionally, two false negative lesions were identified on ^18^F-DCFPyL PET/CT imaging. One lesion was located in the skull and demonstrated local increased bone density without an evident increase in radioactivity distribution. The other lesion was located in the lower segments of the left tibia, which was missed due to the limited imaging range of ^18^F-DCFPyL PET/CT (only covering the head to the thigh roots), and the patient lacked significant lower limb symptoms, so limb scanning was not performed.

Based on patient-level analysis, bone metastasis was diagnosed in 18 out of 32 patients using ^99m^Tc-MDP SPECT/CT imaging. Among them, four were later confirmed to be false positives, while the remaining 14 patients who tested positive for ^99m^Tc-MDP SPECT/CT imaging were confirmed by pathology or BVC to have bone metastatic lesions. Additionally, there were four cases where bone metastases were not detected by ^99m^Tc-MDP imaging, resulting in false negative results. At the lesion-level analysis, 67 lesions showed uptake of ^99m^Tc-MDP according to ^99m^Tc-MDP SPECT/CT imaging. Pathology or BVC confirmed that 61 of them were bone metastases from prostate cancer. The remaining 6 lesions, which included 3 cases of old rib fractures, 2 cases of degenerative changes in the lumbar vertebrae, and 1 case of osteoid osteoma in the proximal femur, were benign bone lesions and represented false positive results. Moreover, 23 bone metastatic lesions did not show obvious uptake of ^99m^Tc-MDP, which were false negative results.

### Comparison of diagnostic efficacy between ^18^F-DCFPyL and ^99m^Tc-MDP

**Figure 1:**
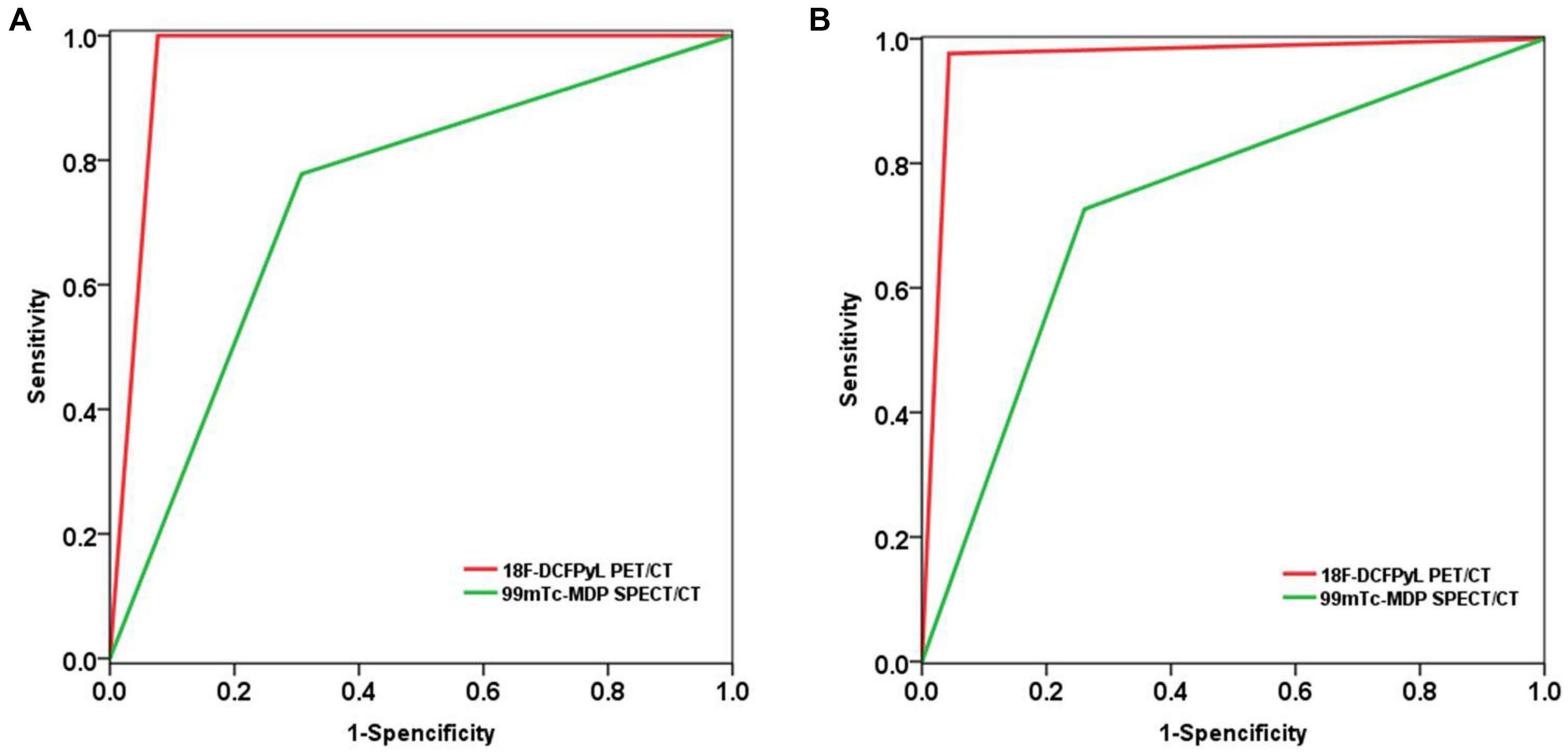
Compare the diagnostic performance of ^18^F-DCFPyL PET/CT and ^99m^Tc-MDP SPECT/CT in detecting bone metastasis of prostate cancer using ROC analysis. **(A)** Based on patient-level analysis; **(B)** Based on lesion-level analysis.

Based on a patient-level analysis, the sensitivity of ^18^F-DCFPyL PET/CT imaging and ^99m^Tc-MDP SPECT/CT bone imaging in diagnosing bone metastases in prostate cancer was 100% (18/18, 95% CI 78.1–100%) and 77.8% (14/18, 95% CI 51.9–92.6%), respectively, and the specificity was 92.3% (12/13, 95% CI 62.1–99.6%) and 69.2% (9/13, 95% CI 38.9–89.6%), respectively. The accuracy was 96.8% (30/31, 95% CI 82.4–99.9%) and 74.2% (23/31, 95% CI 56.5–86.5%), respectively, and the PPV was 94.7% (18/19, 95% CI 71.9–99.7%) and 77.8% (14/18, 95% CI 51.9–92.6%), respectively. NPV was 100% (12/12, 95% CI 69.9–100%) and 69.2% (9/13, 95% CI 38.9–89.6%), respectively. The efficacy of ^18^F-DCFPyL PET/CT imaging in diagnosing bone metastases in prostate cancer was superior to that of ^99m^Tc-MDP SPECT/CT bone imaging (*p* < 0.05), as shown in Table 2. The ROC curve analysis results for the two imaging methods showed that the AUCs were 0.962 and 0.735, respectively, and the AUC for ^18^F-DCFPyL PET/CT imaging was significantly greater than that for ^99m^Tc-MDP SPECT/CT bone imaging (*Z* = 2.168, *p* = 0.015), as shown in Figure 1A.

**Table 2:** Comparison of diagnostic performance indicators for bone metastasis of prostate cancer based on ^18^F-DCFPyL and ^99m^Tc-MDP at the patient level.

	Sensitivity	Specificity	Accuracy	PPV	NPV
^18^F-DCFPyL PET/CT	100%	92.3%	96.8%	94.7%	100%
^99m^Tc-MDP SPECT/CT	77.8%	69.2%	74.2%	77.8%	69.2%
*p* value	*p* < 0.01	*p* < 0.05	*p* < 0.01	*p* < 0.01	*p* < 0.01

Based on a lesion-level analysis, the sensitivity of ^18^F-DCFPyL PET/CT imaging and ^99m^Tc-MDP SPECT/CT bone imaging in diagnosing bone metastases in prostate cancer were 97.6% (82/84, 95% CI 90.9–99.6%) and 72.6% (61/84, 95% CI 61.6–81.5%), respectively, and the specificity were 95.7% (22/23, 95% CI 76.0–99.8%) and 73.9% (17/23, 95% CI 51.3–88.9%), respectively. The accuracy was 97.2% (104/107, 95% CI 91.7–99.4%) and 72.9% (78/107, 95% CI 63.8–80.5%), respectively, and the positive predictive values (PPVs) were 98.8% (82/83, 95% CI 92.5–99.9%) and 91.0% (61/67, 95% CI 80.9–96.3%), respectively. The negative predictive values (NPVs) were 91.7% (22/24, 95% CI 71.5–98.5%) and 42.5% (17/40, 95% CI 27.4–59.0%), respectively. The efficacy of ^18^F-DCFPyL PET/CT imaging in diagnosing bone metastases in prostate cancer was superior to that of ^99m^Tc-MDP SPECT/CT bone imaging (*p* < 0.05), as shown in Table 3. The ROC curve analysis results for the two imaging methods showed that the areas under the curve (AUCs) were 0.966 and 0.733, respectively, and the AUC for ^18^F-DCFPyL PET/CT imaging was significantly greater than that for ^99m^Tc-MDP SPECT/CT bone imaging (*Z* = 3.541, *p* < 0.001), as shown in Figure 1B.

**Table 3:** Comparison of diagnostic performance indicators for bone metastasis of prostate cancer based on ^18^F-DCFPyL and ^99m^Tc-MDP at the lesion level.

	Sensitivity	Specificity	Accuracy	PPV	NPV
^18^F-DCFPyL PET/CT	97.6%	95.7%	97.2%	98.8%	91.7%
^99m^Tc-MDP SPECT/CT	72.6%	73.9%	72.9%	91.0%	42.5%
*p* value	*p* < 0.01	*p* < 0.01	*p* < 0.01	*p* < 0.01	*p* < 0.01

## Discussion

The skeleton is the most common site of distant metastasis in prostate cancer, and bone-related events (SREs) caused by metastasis increase the risk of death in prostate cancer patients by 28% (13). In recent years, PSMA PET imaging technology has developed rapidly. Currently, the FDA has approved two PSMA-based PET/CT tracers, ^68^Ga-PSMA-11 and ^18^F-DCFPyL, for clinical diagnosis of prostate cancer patients. Compared to ^68^Ga-PSMA-11, ^18^F-DCFPyL has several advantages, such as higher drug yield, better image resolution, and easier commercial operation. Studies have shown that ^18^F-DCFPyL has higher SUV values and tumor-to-background ratios than ^68^Ga-PSMA-11 in the diagnosis of prostate cancer primary lesions, especially for the diagnosis of smaller lesions (14). Therefore, ^18^F-DCFPyL has rapidly gained widespread popularity in routine clinical diagnosis of prostate cancer patients. However, there is still limited clinical data on the use of ^18^F-DCFPyL PET/CT imaging to diagnose bone metastasis. In a preliminary study of eight patients, Rowe et al. found that ^18^F-DCFPyL PET/CT imaging could detect occult bone metastases that conventional methods such as X-ray and CT cannot detect (15). In a prospective study of 130 patients with biochemical recurrence of prostate cancer in OSPREY, the diagnostic sensitivity and positive predictive value of ^18^F-DCFPyL PET/CT for bone metastases were 96.8 and 81.6%, respectively. However, this study did not further compare its diagnostic performance with traditional imaging methods (16).

The selected patients in this study were primarily categorized as intermediate to high risk based on their SPA levels and Gleason scores. A relatively high proportion of bone metastasis was observed, accounting for 58.06% (18/31) of cases, with the pelvis and lumbar vertebrae being the most frequently affected locations. This may be attributed to the abundance of red bone marrow in the pelvis and lumbar vertebrae, as well as the existence of the Batson venous plexus, which has rich blood flow and is more prone to bone metastasis. When analyzed on a patient basis, the sensitivity and specificity of ^18^F-DCFPyL PET/CT were 100.0 and 92.3%, respectively, while those of ^99m^Tc-MDP SPECT/CT were 77.8 and 69.2%. When analyzed on a lesion basis, the sensitivity and specificity of ^18^F-DCFPyL PET/CT were 97.6 and 95.7%, respectively, while those of ^99m^Tc-MDP SPECT/CT were 72.6 and 73.9%, respectively. The results of this study demonstrated that the diagnostic performance of ^18^F-DCFPyL PET/CT was similar to previous studies (17). However, the sensitivity and specificity of ^99m^Tc-MDP SPECT/CT bone scanning in this study were slightly lower than those reported in previous studies, possibly due to the fact that only SPECT/CT imaging was performed on the suspicious areas based on the whole-body planar imaging, and not whole-body computed tomography imaging on all patients (11). Among the 31 patients included in our study, 4 patients showed no obvious uptake of ^99m^Tc-MDP throughout the body, but were accurately diagnosed with bone metastasis using ^18^F-DCFPyL PET/CT, which allowed for more accurate tumor staging and effective optimization of treatment plans. Of the 84 confirmed metastatic lesions in this study, 23 lesions that were negative on ^99m^Tc-MDP SPECT/CT bone scanning were accurately identified by ^18^F-DCFPyL PET/CT imaging. These lesions did not show any significant changes in bone density or morphology on computed tomography scanning. This may be due to the fact that early bone metastases only cause changes in red bone marrow, and bone destruction and neogenesis are not yet significant. Due to the limitations of the principle of ^99m^Tc-MDP uptake, bone scanning cannot detect bone metastases at this stage, while ^18^F-DCFPyL, by specifically binding with PSMA on cancer cells, can detect these early metastatic lesions, which provides a significant advantage in their discovery (18).

In this study, two false negative lesions were detected by ^18^F-DCFPyL PET/CT imaging. One of them was found in a patient with castration-resistant prostate cancer (CRPC), where the lesion in the skull showed low uptake of ^18^F-DCFPyL, possibly due to low tumor cell activity or quantity after clinical treatment (19, 20). The other false negative lesion was located in the middle and lower part of the left tibia, which was mainly due to the limited routine imaging range of ^18^F-DCFPyL PET/CT. Both lesions showed obvious radiopharmaceutical concentration in the ^99m^Tc-MDP SPECT/CT bone imaging. Although these two patients were not affected in the final clinical staging and treatment plan due to the presence of other bone metastases, it suggests that a comprehensive diagnosis should be made by combining multiple imaging results, biochemical indicators, clinical history, and signs to avoid misdiagnosis or missed diagnosis of prostate cancer bone metastases. Furthermore, in this study, one false positive lesion was detected by ^18^F-DCFPyL PET/CT imaging, which was located in the right sacroiliac joint and was confirmed by pathological biopsy as degenerative joint disease. Although ^18^F-DCFPyL PET/CT imaging is a specific imaging method for targeting prostate cancer and its metastatic lesions, previous studies have shown that ^18^F-DCFPyL uptake can also occur to varying degrees in non-tumor lesion sites such as neovascularization, sympathetic ganglia, and bone degenerative lesions, which may be the main reason for false positive findings in such lesions (21).

This study has certain limitations. Firstly, it is a retrospective clinical study with fairly strict inclusion criteria, requiring patients to undergo both ^18^F-DCFPYL PET/CT imaging and ^99m^Tc-MDP SPECT/CT bone imaging within a short period of time (<30 days), and no changes in clinical treatment are allowed during the interval between the two examinations. This led to a relatively small number of patients included in this study, which may have resulted in certain biases in the research findings. Secondly, there is a lack of pathological gold standards for the diagnosis of bone metastases in this study, with most final judgments being made based on BVC results. Although all suspicious bone metastases were further evaluated by BVC, the presence of additional bone metastases cannot be completely ruled out.

## Conclusion

In summary, our research demonstrates that ^18^F-DCFPyL PET/CT shows superior sensitivity, specificity, and accuracy in detecting bone metastases in patients with prostate cancer when compared to ^99m^Tc-MDP SPECT/CT bone scanning. While performing ^18^F-DCFPyL PET/CT for all prostate cancer patients may not be cost-effective, it remains a favorable option for patients at intermediate or high risk, even in cases where bone scanning and routine imaging yield negative results.

## Data availability statement

The raw data supporting the conclusions of this article will be made available by the authors, without undue reservation.

## Ethics statement

The studies involving human participants were reviewed and approved by Ethic committee of China-Japan Union Hospital. The patients/participants provided their written informed consent to participate in this study.

## Author contributions

BC and QW conceived and design the study, which were proofed by BC. XH, BJ, YC, and MZ collected and analyzed the data. XH wrote the manuscript. All authors contributed to the article and approved the submitted version.

## Funding

This research was supported by Research Fund of Science and Technology Department of Jilin Province (No. 20210101445JC) and The Research Fund of Education Department of Jilin Province (No. JJKH20190080KJ).

## Conflict of interest

The authors declare that the research was conducted in the absence of any commercial or financial relationships that could be construed as a potential conflict of interest.

## Publisher’s note

All claims expressed in this article are solely those of the authors and do not necessarily represent those of their affiliated organizations, or those of the publisher, the editors and the reviewers. Any product that may be evaluated in this article, or claim that may be made by its manufacturer, is not guaranteed or endorsed by the publisher.
